# Supplementary material for: The impact of resident involvement and postgraduate year on head and neck surgery for obstructive sleep apnea
Source: PLoS One. 2025 Jan 13;20(1):e0317381. doi: 10.1371/journal.pone.0317381 (PMC11729955; doi:10.1371/journal.pone.0317381)
Supplement: Attachment — Submitted filename: Response To Reviewers_4thRound.docx [file pone.0317381.s001.docx]

Dear Editors and Reviewers,

Thank you for inviting our team to submit a revised draft of our manuscript entitled, “The impact of resident involvement and postgraduate year on head and neck surgery for obstructive sleep apnea” (PONE-D-24-20350) to *PLOS ONE*. We appreciate the effort and time that the reviewers dedicated in providing their insight and helpful feedback. We agree that this extra effort further strengthens our manuscript. We have incorporated the suggestions from the incite that was provided by the reviewers. We anticipate that the edits and responses that we have shared below addresses the concerns and recommendations you and the reviewers have provided.

To aid in the facilitation of your review of the revisions, the following is a point-by-point response to the questions and comments delivered in your revision letter. To facilitate readthrough, we have separated each question/comment using the header COMMENT and then replied to each comment via the header RESPONSE.

Thank you once again for your time and consideration.

**COMMENT:** We note that your "Manuscript and Cover Letter" files are duplicated on your submission. Please remove any unnecessary or old files from your revision, and make sure that only those relevant to the current version of the manuscript are included.

**RESPONSE:** All unnecessary / old files have been removed from the submission. Only the current versions of the manuscript and cover letter have been included. We apologize for any confusion caused by this action.

**COMMENT:** We understand that you have updated your data availability statement to read as follows: "The data is part of the ACS-NSQIP PUF database maintained by the American College of Surgeons. The data is available free of charge from the American College of Surgeons for members of hospitals participating in the NSQIP. Data access queries for non-members can be directed to the Corresponding Author, Mihai Bentan ([bentanm@vcu.edu](mailto:bentanm@vcu.edu)).

Public disposition of this data would breach the Data Use Agreement (and therefore copyright laws) as imposed by the American College of Surgeons, as detailed in the Participant Use Data File ‘Access to Chapter 4’ section: “Chapter 4 (for both Essential and Procedure Targeted data) contains proprietary ACS NSQIP programmatic information. Your use of Chapter 4 is restricted to clarifying definitions for your PUF-based research purposes. The contents of Chapter 4 shall not be shared, quoted, distributed, or disseminated in any published papers, reports, manuscripts, etc. Limited paraphrasing of Chapter 4 definitions, to provide methodological clarity in research publications, is permissible. Any use or redistribution of Chapter 4 for commercial purposes is strictly prohibited.” Therefore, given these copyright laws, we are unable to provide the data underlying our finals in any of the aforementioned methods (a-c) so as to avoid violating copyright laws. However,
individuals/readers interested seeking permission to use the data may follow contact the ACS who will process a request, as detailed in the following: “Permission to use and disclose the PUF is granted from the ACS NSQIP to each Data Recipient. The Data Recipient must be an employee of the participating Hospital. Intentional misrepresentation of employment at the Hospital by the Data Recipient will void this Agreement, prohibit use of the PUF by the Data Recipient, and result in legal action taken by the ACS NSQIP. The ACS NSQIP also reserves the right to deny access to the PUF at its discretion.” Further information regarding all of this information can be found at the following link: <https://www.facs.org/media/ezaj2g5p/datauseagreementacsnsqippuf.pdf>

The data is owned by a third-part institution (ACS NSQIP) and therefore they cannot be shared by any of the authors of this study. The following URL: <https://www.facs.org/quality-programs/data-and-registries/pediatric/participant-use-data-file/participant-use-request-form/> should be utilized to identify the appropriate non-author contact for the location/state in which the requesting individual resides. Other individuals are able to access this data in the same manner that these authors have. We report that these authors did not have any specific access privileges that others would not have had."

However, this statement does not provide non-author contact information for requesting these data. Please include a non-author email address in this statement.

**RESPONSE:** Thank you for bringing this to our attention. The following non-author email address, [acsnsqip@facs.org](mailto:acsnsqip@facs.org), may be utilized for data requests. This information has been added to the data availability statement, which now reads as follows:

The data is part of the ACS-NSQIP PUF database maintained by the American College of Surgeons. The data is available free of charge from the American College of Surgeons for members of hospitals participating in the NSQIP. Data access queries for non-members can be directed to the Corresponding Author, Mihai Bentan ([bentanm@vcu.edu](mailto:bentanm@vcu.edu)).

Public disposition of this data would breach the Data Use Agreement (and therefore copyright laws) as imposed by the American College of Surgeons, as detailed in the Participant Use Data File ‘Access to Chapter 4’ section: “Chapter 4 (for both Essential and Procedure Targeted data) contains proprietary ACS NSQIP programmatic information. Your use of Chapter 4 is restricted to clarifying definitions for your PUF-based research purposes. The contents of Chapter 4 shall not be shared, quoted, distributed, or disseminated in any published papers, reports, manuscripts, etc. Limited paraphrasing of Chapter 4 definitions, to provide methodological clarity in research publications, is permissible. Any use or redistribution of Chapter 4 for commercial purposes is strictly prohibited.” Therefore, given these copyright laws, we are unable to provide the data underlying our finals in any of the aforementioned methods (a-c) so as to avoid violating copyright laws. However,
individuals/readers interested seeking permission to use the data may follow contact the ACS who will process a request, as detailed in the following: “Permission to use and disclose the PUF is granted from the ACS NSQIP to each Data Recipient. The Data Recipient must be an employee of the participating Hospital. Intentional misrepresentation of employment at the Hospital by the Data Recipient will void this Agreement, prohibit use of the PUF by the Data Recipient, and result in legal action taken by the ACS NSQIP. The ACS NSQIP also reserves the right to deny access to the PUF at its discretion.” Further information regarding all of this information can be found at the following link: <https://www.facs.org/media/ezaj2g5p/datauseagreementacsnsqippuf.pdf>

The data is owned by a third-part institution (ACS NSQIP) and therefore they cannot be shared by any of the authors of this study. The following URL: <https://www.facs.org/quality-programs/data-and-registries/pediatric/participant-use-data-file/participant-use-request-form/> should be utilized to identify the appropriate non-author contact for the location/state in which the requesting individual resides. Other individuals are able to access this data in the same manner that these authors have. We report that these authors did not have any specific access privileges that others would not have had. Data requests may be performed using the aforementioned URL link or via the following email address: [acsnsqip@facs.org](mailto:acsnsqip@facs.org).
